# Radioembolisation in Europe: A Survey Amongst CIRSE Members

**DOI:** 10.1007/s00270-018-1982-4

**Published:** 2018-05-08

**Authors:** Margot T. M. Reinders, Etienne Mees, Maciej J. Powerski, Rutger C. G. Bruijnen, Maurice A. A. J. van den Bosch, Marnix G. E. H. Lam, Maarten L. J. Smits

**Affiliations:** 1 Department of Radiology and Nuclear Medicine, University Medical Centre Utrecht (UMCU), House Post No. E01.132, P.O. Box 85500, 3508 GA Utrecht, The Netherlands; 2 Utrecht University (UU), P.O. Box 80125, 3508 TC Utrecht, The Netherlands; 3 Department of Radiology and Nuclear Medicine, Otto-von-Guericke University, Magdeburg, Germany

**Keywords:** Radioembolisation, Interventional oncology, Liver, Yttrium

## Abstract

**Introduction:**

Radioembolisation of liver tumours demands many choices from the physician regarding planning of treatment and subsequent follow-up.

**Methods:**

An online questionnaire was distributed amongst all members of the Cardiovascular and Interventional Radiological Society of Europe (CIRSE) to investigate the current state of radioembolisation practice.

**Results:**

The survey was completed by 60 centres. The increasing number of radioembolisation procedures may reflect that radioembolisation is increasingly recognised as a valuable treatment option in European cancer guidelines. Imaging modalities play an important role in decision making. Furthermore, there seems to be a trend towards less coil-embolisation of non-target vessels. In addition, type of microsphere, model for dose calculation, complications and future developments are evaluated in this article.

**Conclusions:**

This survey provides insight into the current state of radioembolisation practice across Europe.

**Electronic supplementary material:**

The online version of this article (10.1007/s00270-018-1982-4) contains supplementary material, which is available to authorized users.

## Introduction

Radioembolisation (RE) is a catheter-based internal radiation therapy with high radiation doses aimed at the tumours, while having a minimal effect on the surrounding non-tumorous liver tissue [1]. This treatment relies on the fact that hepatic malignancies derive most of their blood supply from the hepatic artery, while the non-tumorous tissue is mainly supplied via the portal vein [2]. Due to this mechanism, a tumouricidal dose can be obtained while sparing most of the non-tumorous liver tissue [1, 3].

Radioembolisation requires close collaboration of a multidisciplinary team, in which intervention-radiologists and nuclear physicians take the lead together. It is a complex image-guided procedure and can be performed in a large variety of ways. Operators have to make choices ranging from the type of microspheres, type of catheters, use of imaging modalities to methods for calculating the activity. A survey from 2011 (published in 2012) showed that there are many differences in how radioembolisation is performed across 28 centres in Europe [4]. Now, about 7 years later, the radioembolisation community has grown and there have been technical and strategic changes in the field, whereas some centres prefer coiling of (several) arteries, while others do not. There also seems to be a discrepancy between lung shunt correction implementation in the centres and the instructions for use provided by the medical device companies. Furthermore, cone-beam CT (also known as C-arm CT) is introduced as a tool in the radioembolisation procedure and a significant increase in the use of SPECT–CT is seen. To investigate how these changes have impacted daily practice in European centres and what differences exist between centres, a questionnaire was spread amongst all members of the Cardiovascular and Interventional Radiological Society of Europe (CIRSE).

## Methods

### The Questionnaire

 The questionnaire consisting of 25 questions was developed in analogy to the questionnaire developed by Powerski et al. [4]. The questions from the study by Powerski et al. were updated, and new questions were added (Table 1). The questions were divided into five categories, questions concerning (1) the centre performing radioembolisation, (2) patient work-up, (3) patient treatment, (4) patient aftercare, and (5) future developments. In order to facilitate logistics, to reach a large audience and to reduce missing data and handling time, the survey was placed on a dedicated website. The website was created in WordPress using the Gravity-forms plugin (Rocketgenius, Inc.) by the Multimedia team of the Imaging Division of University Medical Centre Utrecht (UMCU).

**Table 1:** Questions and answers as presented in the survey of 2017

	Questions	Answers
1	What is the name and location of your treatment centre?	Name and City, Country
2	In what year did your institution start with radioembolisation?	
3	On estimation, how many radioembolisations were performed at your department in …?	2014: … 2015: … 2016: … 1–10/11–25/26–50/> 100
4	How frequent do you encounter the following indications for hepatic radioembolisation at your department? (average number of patients per year)	0–5/6–10/11–25/26–50/> 50
	Hepatocellular carcinoma/cholangiocarcinoma/colorectal carcinoma metastasis/breast cancer metastasis/neuroendocrine tumour metastasis/other	
5	What kind of microspheres do you use for radioembolisation?	Resin (SIR-Spheres)/glass (TheraSpheres)/other
6	(a) What imaging modality is most commonly used for pretreatment staging of disease at your institution?	CT/MRI/PET–CT/other
	(b) Do you assess the arterial liver anatomy prior to angiography?	No/Yes with MRA/Yes with CTA/other
7	Are the following conditions contraindications to perform radioembolisation at your institute? Biliodigestive anastomosis/biliary stenting/biliary drainage/condition after papillotomy/arterio-portal shunt/previous bland or chemo-embolisation/complete portal vein thrombosis/lobar portal vein thrombosis/tumour burden > 70%/primary tumour not resected/extrahepatic disease/ascites drainage	Always a contraindication/most of the time a contraindication/undecided/most of the time not a contraindication/never a contraindication
8	What kind of imaging do you use to evaluate 99m Tc-MAA distribution?	Planar/SPECT/SPECT–CT/other
9	What is/are the main reason(s) for you to perform evaluation with 99m Tc-MAA before radioembolisation	Lung shunt assessment/extrahepatic deposition assessment (e.g. intestines)/intrahepatic assessment (tumour targeting)/other
10	When do you consider lung shunting as contraindication?	Shunt volume percentage …/estimated absorbed radiation dose …/other
11	On average, how many patients (%) do you exclude due to too high lung shunting?	0–1/2–5/6–10/11–25/> 25%
12	How many of all patients (%) receive dose reduction due to increased lung shunting?	0–1/2–5/6–10/11–25/> 25%
13	(a) What method do you use to calculate the amount of activity of resin spheres to be injected?	Empirical method/Body Surface Area (BSA) method/partition model/other
	(b) What method do you use to calculate the amount of activity of glass spheres to be injected?	Empirical method/MIRD model/other
14	(a) Which arteries, if any, do you embolise during diagnostic angiography? Gastroduodenal artery Right gastric artery Cystic artery Other	Never/incidentally/sometimes/most of the time/always
15	What kind of medication do you prescribe? (a) Pretreatment (b) During treatment (c) Post-treatment	Steroids/opioids/NSAIDs/paracetamol/metamizole/anti-emetics/proton-pump inhibitor/other
16	What kind of microcatheter do you use for the administration of spheres?	Standard microcatheter (e.g. Progreat^®^, Cantata^®^, Direxion™)/anti-reflux microcatheter (e.g. Surefire^®^ Infusion System, Balloon Catheter)/other
17	Do you check whether the position/location of the catheter is the same during the actual treatment as it was during the MAA procedure? If yes, how?	No, I don’t check whether the position is the same/yes, I try to recall the injection position from the 99mTc-MAA injection/yes, I try to closely mimic the injection position from the 99mTc-MAA injection by visually comparing the positions on angiography/yes, but in a different way
18	What is your preferred sphere administration technique in case of bilobar manifestation of tumour?	Whole liver (bilobar) infusion in a single session via proper hepatic artery/left and right hepatic artery in a single session/sequential left/right radioembolisation with a time gap of… weeks/other
19	For what purpose do you use C-arm CT (e.g. cone-beam CT) for radioembolisation?	C-arm CT is not used at all/for extrahepatic deposition assessment/to check tumour coverage/for volumetric analysis/calculation of activity needed/other
20	Do you use post-treatment imaging to evaluate microsphere distribution?	No/yes, 90Y–PET–CT/yes, 90Y–Bremsstrahlung SPECT/yes, 90Y–Bremsstrahlung SPECT–CT/Yes, other
21	At your institute, what percentage of patients treated with radioembolisation receive a 2nd or 3rd treatment with radioembolisation?	0–1/2–5/6–10/11–25/> 25%
22	At your institute, what imaging modality for tumour status is most commonly used in radiological follow-up?	CT/MRI/PET/PET–CT/other
23	On estimation, how frequent (% of all patients) do you encounter the following complications in radioembolisation patients? Radiation pneumonitis/gastrointestinal complications/pancreatic complications/radioembolisation induced liver disease (RILD)/bile duct complications/cholecystitis/abscess/other	0–1/2–5/6–10/11–25/> 25%
24	Which of the following (potential) developments could improve radioembolisation treatment in your practice? Better insight into the distribution of the microspheres in the liver The possibility to adapt the distribution of the microspheres during the treatment based on real-time imaging feedback on the dose distribution Improved options to calculate the amount of activity to be injected Improved catheter designs	Strongly disagree/disagree/neutral/agree/strongly agree
25	Are there any other (potential) developments you see to improve radioembolisation treatment in your practice?	

The welcome page displayed instructions for filling out the survey. Since radioembolisation is a treatment leaning on both interventional radiology and nuclear medicine, respondents were advised to seek help from a colleague interventional radiologist or nuclear medicine physician to answer questions beyond their own expertise.

Respondents could start the survey and finish at a later point in time as it was possible to meanwhile save the results. In order to obtain a complete data set, most questions could not be left blank. Where possible, multiple-choice questions were used for ease and efficiency. Most questions had an option to leave comments, in case the desired option was not listed or additional information was necessary.

### Spreading the Questionnaire

All CIRSE members received an invitation by e-mail to fill out the questionnaire on April 13th of 2017. This e-mail contained the uniform resource locator (URL) and password needed to access the questionnaire. A reminder was sent to all CIRSE members 3 weeks after the first invitations were sent to increase the response rate.

### Statistics and Analysis

Responses were collected and then analysed in R for Windows (version 3.4.1.). When a question was left blank by a certain centre, the response ‘Unknown’/‘Undecided’/‘No’ (depending on the question) was added and used in the analysis. Descriptive statistics (mean and percentages) were used to display the results. Occasionally, the percentages described do not add up to 100% as there were questions where multiple options could be checked.

## Results

**Fig. 1:**
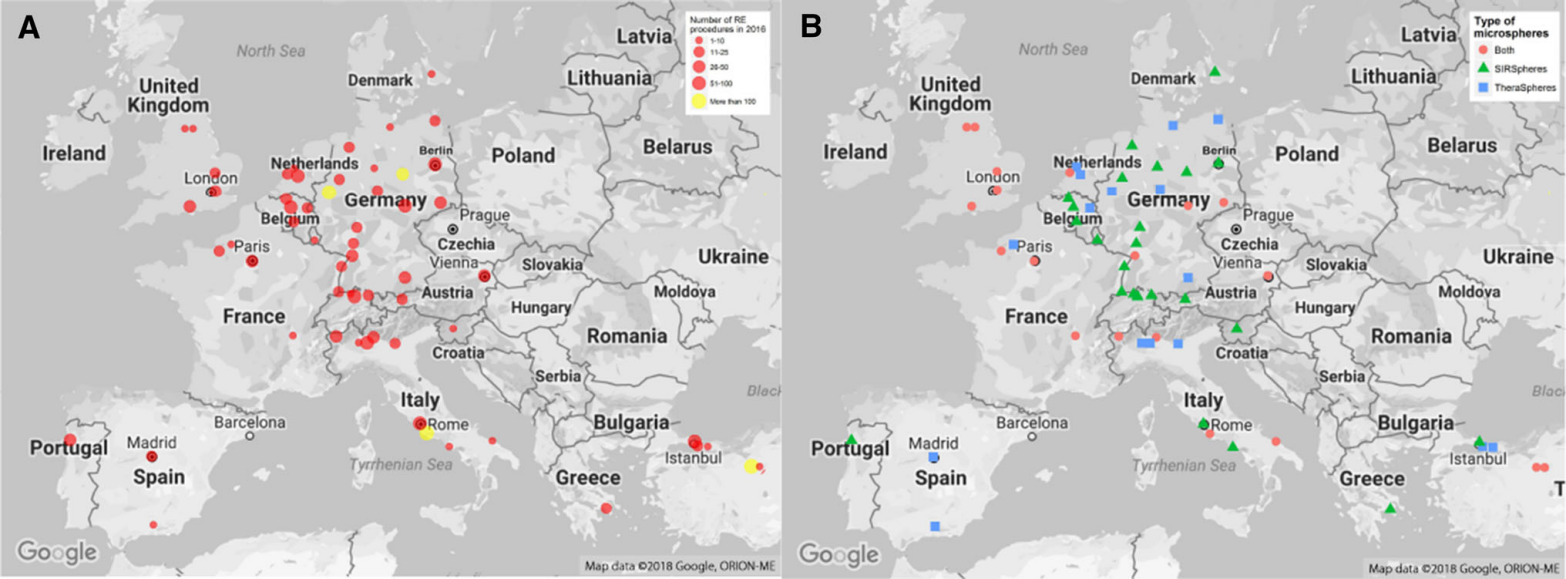
**A** Geographical representation of number of radioembolisation procedures per centre in 2016 (Q3). **B** Geographical representation of the type of microspheres used in the 60 participating centres (Q5)

Seventy-one centres completed the questionnaire, of which 37 centres filled it out after the first round of invitations. The remaining 34 centres responded after the reminder. Responses from centres outside of Europe were excluded (*n* = 8), so were centres that did not perform radioembolisation procedures in 2014, 2015 and/or 2016 (*n* = 3). Therefore the included centres were deemed representative of the radioembolisation centres in Europe. Not all centres answered all 25 questions altogether. In total, the answers of eight questions (questions 2, 7, 8, 12, 13, 19, 20 and 23) were not complete. However, the results of these questions were based on at least 57 responses (Fig. 1A and Tables S1, S2).

### Demographics

**Fig. 2:**
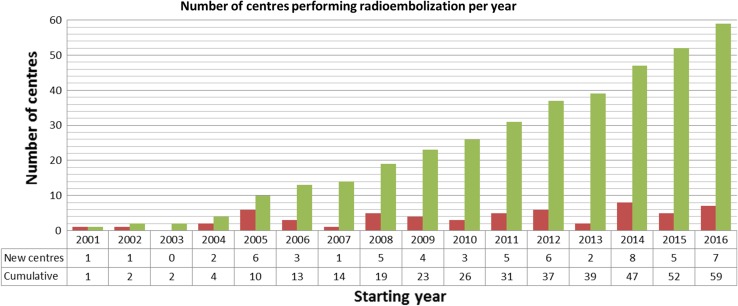
Number of centres starting to perform radioembolisation per year. Please note that these numbers only represent the centres that participated in this survey and one centre did not answer this question (Q2)

Based on the survey data, there seems to be a steady rise in number of centres performing radioembolisation with 1 centre starting to perform radioembolisation in 2001 and 7 centres in 2016. Cumulatively, 60 centres in Europe perform radioembolisation, which is shown in Fig. 2 (one centre did not specify the start year of radioembolisation; therefore, only 59 data points are available). Regarding the number of procedures, the majority of centres (80%) performed between 1 and 50 radioembolisation procedures in 2016. The number of centres performing more than 25 treatments per year has increased over the last 3 years from 20 centres in 2014 to 25 centres in 2016 (Fig. 1A and Fig. S1A).

**Fig. 3:**
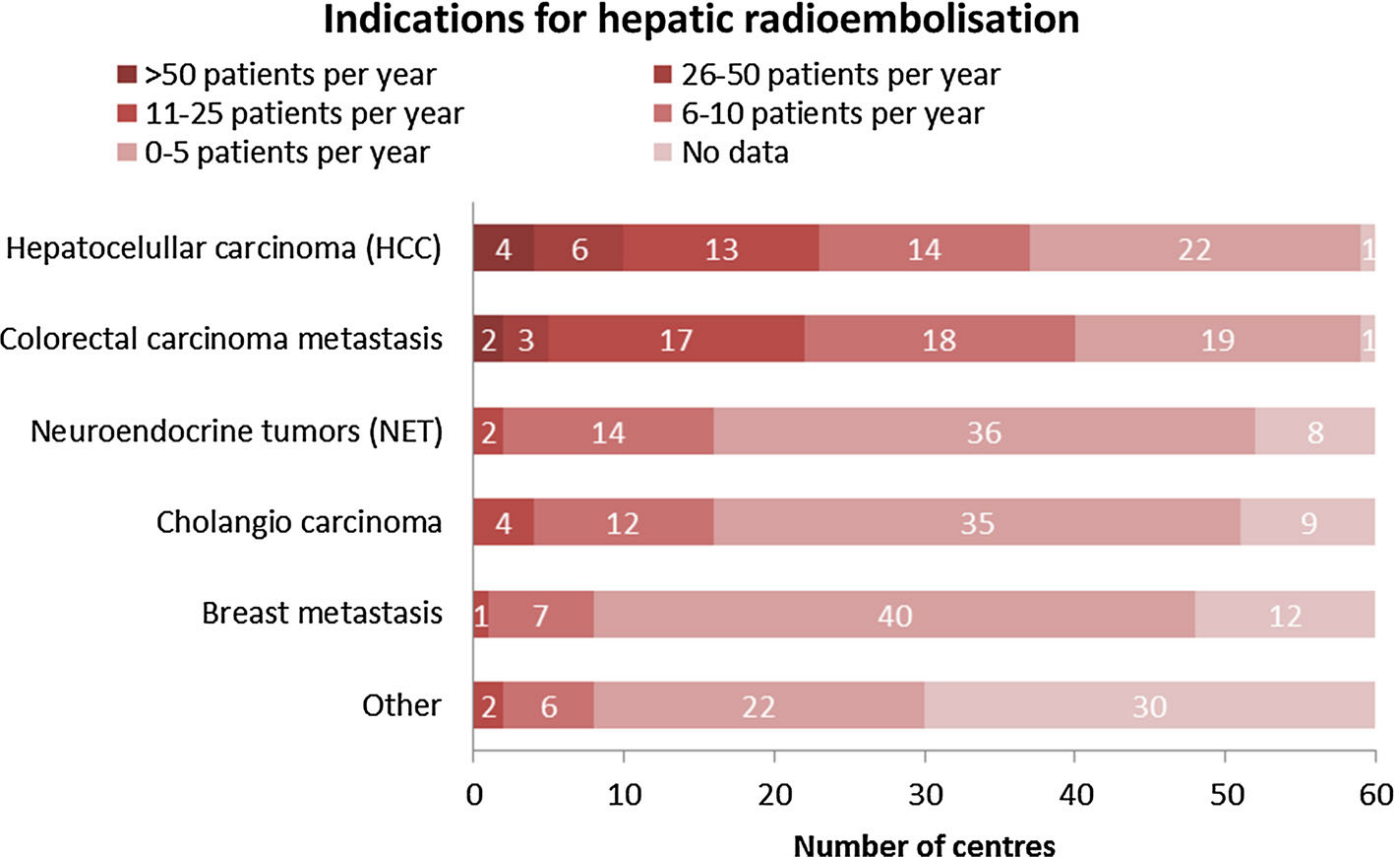
Heat map representing number of patients per tumour type per centre per year (Q4)

Hepatocellular carcinoma (HCC) and colorectal carcinoma metastases (mCRC) were the most frequent tumour types treated with radioembolisation as 15 centres treat more than 25 patients per year with these tumour types. Cholangiocarcinoma (73% of the centres treats 0–5 patients per year), breast cancer metastases (87%; 0–5 patients per year), and neuroendocrine tumour metastases (73%; 0–5 patients per year) were less common (Fig. 3). Furthermore, there were a few centres that use radioembolisation for pancreas cancer and (ocular) melanoma amongst other tumour types.

Forty percent (40%) of the centres use resin microspheres (SIRSpheres^®^; SIRTeX, North Sydney, Australia) only, whereas 27% only use glass microspheres (TheraSpheres^®^; BTG, Ottawa, Ontario, Canada) and approximately 33% use both resin and glass microspheres (Fig. 1B and Table S1). The centres that use both types of microspheres use glass microspheres mainly to treat HCC, while resin microspheres are predominantly used for other tumour types. In addition, several centres mentioned that the choice for a certain type of microsphere was influenced by national reimbursement policies.

### Patient Work-Up

**Fig. 4:**
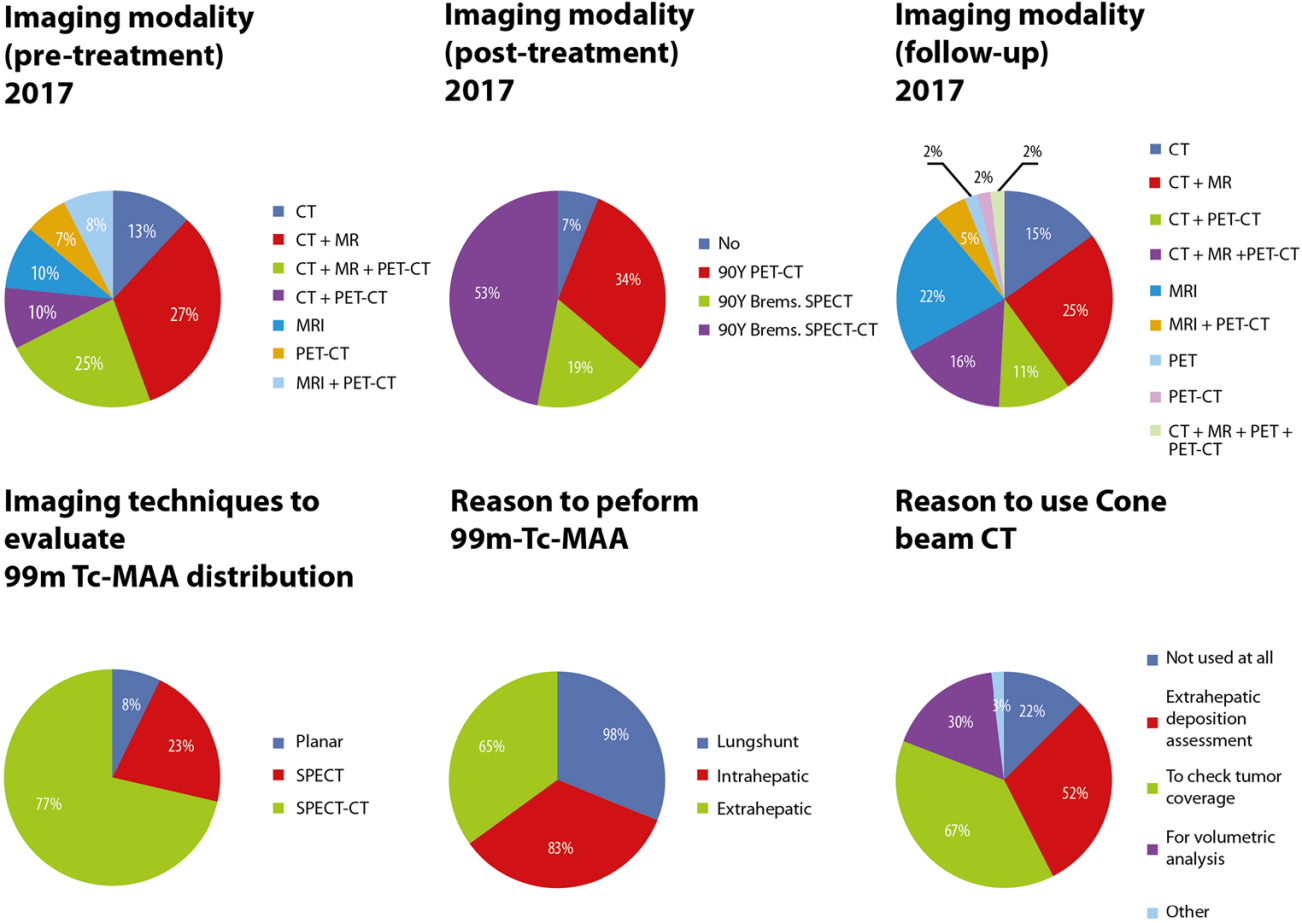
Pie charts regarding imaging techniques used in radioembolisation treatment (Q6a, Q8, Q9, Q19, Q20 and Q22)

In most cases, a combination of imaging modalities is used for pretreatment staging of disease. CT (75%) was most commonly used, closely followed by MRI (70%) and lastly PET–CT (50%) (Fig. 4 and Table S2). Almost 80% of the participating centres assessed arterial liver anatomy prior to angiography, using CTA in 72% versus MRA in 7% of the centres. The remaining centres (22%) did not assess the arterial liver anatomy prior to angiography (Table S2 and Figure S4).

**Fig. 5:**
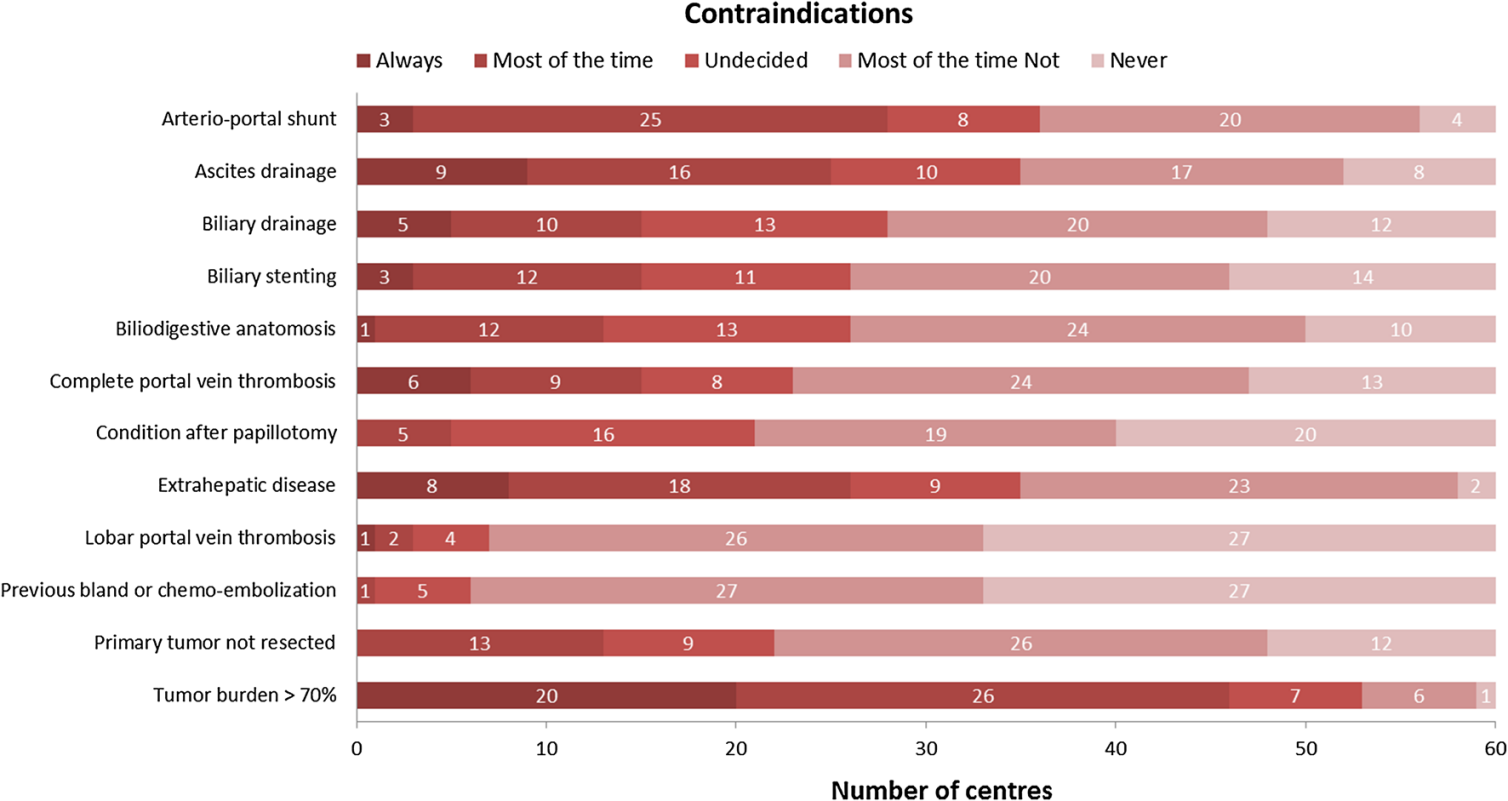
Heat map of the conditions that centres marked as a contraindication or not (Q7)

Tumour burden > 70% is a dominant contraindication in the majority of centres. Biliary issues (e.g. anastomoses, stents, drains, papillotomy), previous bland- or chemo-embolisation, lobar and complete portal vein thrombosis and an unresected primary tumour were no contraindications to perform radioembolisation in most centres. There seems to be no consensus on whether or not arterio-portal shunting, ascites drainage and extrahepatic disease are contraindications (Fig. 5 and Table S2).

SPECT–CT is the main modality to evaluate Technetium-99 m macroaggregated albumin (^99m^Tc-MAA) distribution (77%), besides planar imaging (8%) and SPECT (23%). For most centres, lung shunt assessment is the main reason to perform evaluation with ^99m^Tc-MAA before radioembolisation (98%), followed by extrahepatic deposition assessment (83%), and intrahepatic distribution assessment (65%) (Fig. 4).

**Fig. 6:**
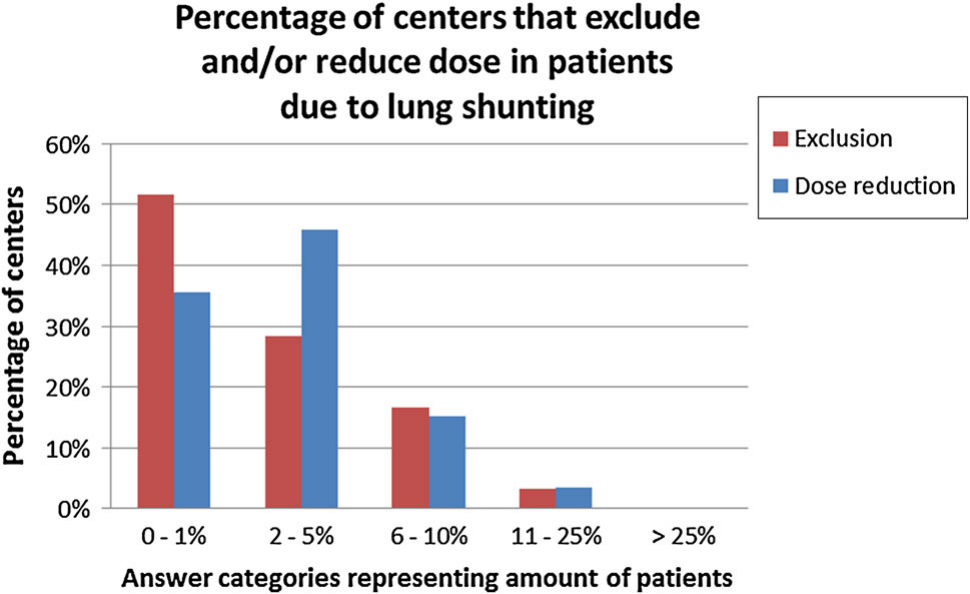
Percentage of centres that exclude and/or reduce dose in patients due to lung shunting (Q11 and Q12)

Lung shunting is the main reason to exclude patients from treatment (82%). Of these centres the majority considered a lung shunt percentage higher than 20% a contraindication (spread between 5 and 50%). Twenty-three percent of the centres used the estimated absorbed radiation dose to the lung instead, with a cut-off dose of 30 Gy (spread between 7 and 50 Gy) (Table S1). Exclusion of patients because of lung shunting is seen in 48% of the centres excluding between 1 and 25% of patients for this reason. Activity reduction is seen in 46% of the centres that reduce the activity in 2–5% of the patients for lung shunting (Fig. 6).

The body surface area (BSA) method was the most commonly used method for calculating the amount of activity of resin microspheres to be injected (normal BSA in 75%, modified BSA in 5%) followed by the partition model method (36%) and the empirical method (16%). For glass microspheres, most centres used the Medical Internal Radiation Dose (MIRD) model (59%) or the empirical method (24%) (Figure S4).

**Fig. 7:**
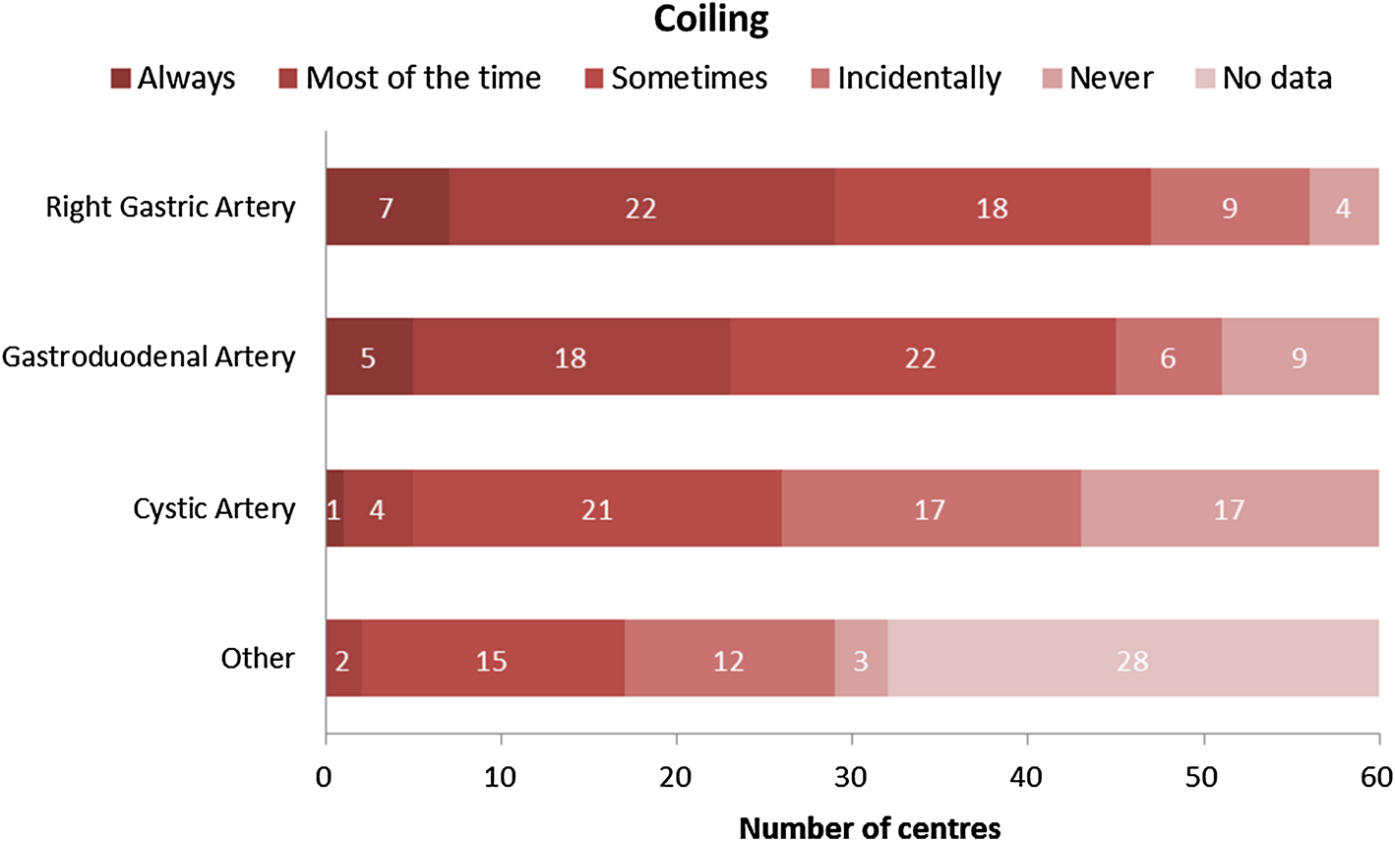
Heat map of frequency of arteries that are coiled by participating centres (Q14)

The degree of coil-embolisation of non-target vessels is highly variable between centres (Fig. 7). The right gastric artery (RGA) is coil-embolised most often (79%). The gastroduodenal artery (GDA) was embolised quite regularly as well (75%). The vast majority of centres (92%) does not coil the cystic artery (CA). Depending on tumour type and vascularity of the tumour other arteries are incidentally coiled.

### Treatment

**Fig. 8:**
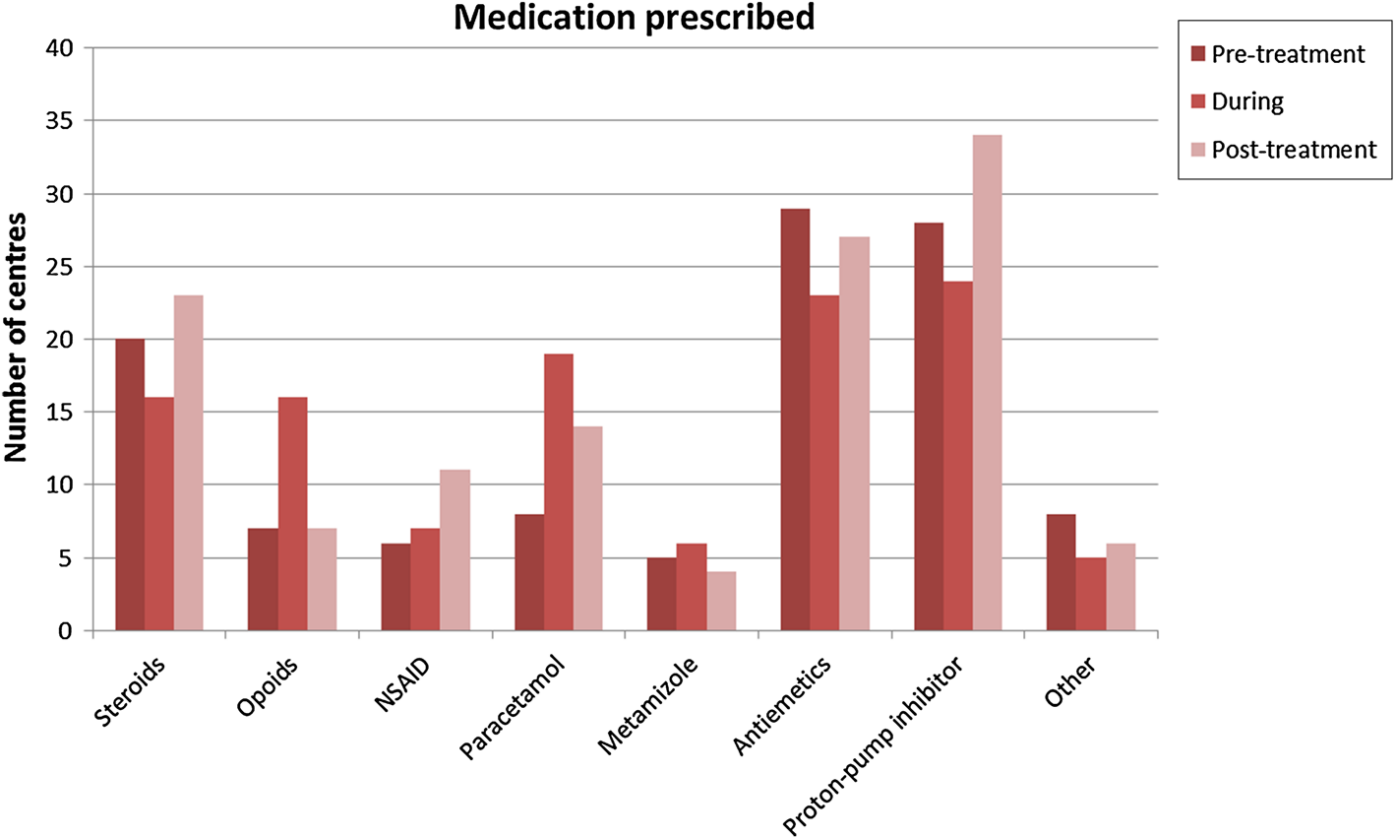
Number of centres that prescribe certain medication in pretreatment, during treatment and post-treatment (Q15)

Prescribed medicine before treatment, during treatment and after treatment included anti-emetics and proton-pump inhibitors. Steroids were also often prescribed before and after treatment. During treatment paracetamol and opioids were given in most centres (Fig. 8 and Table S1).

The vast majority of centres (91%) tried to closely mimic the injection position from the ^99m^Tc-MAA injection during the actual treatment by visually comparing the position. Five percent of the centres did not check at all whether the position during the actual treatment was the same as during the ^99m^Tc-MAA injection. In case of bilobar manifestation of tumour, 55% of the centres choose a sequential left/right radioembolisation with, in most cases, a time gap of 4–6 weeks (Table S1 and Figure S4). Microsphere administration in the left and right hepatic artery in a single session was preferred by 38% of centres. Only 5% of centres performed whole liver (bilobar) infusion in a single session via the proper hepatic artery. Repeated radioembolisation on the same part of the liver is quite common, with two-thirds of centres performing repeated radioembolisation in at least 2% of patients (Figure S4).

Cone-beam CT is not used at all by 22% of centres. Most of the centres that do use it, use it for multiple reasons: to check tumour coverage (67%), extrahepatic deposition assessment (52%), and volumetric analysis (30%) (Fig. 4).

### Aftercare

Seven percent of all centres do not use post-treatment imaging to evaluate microsphere distribution. The other centres often use multiple imaging modalities: Bremsstrahlung SPECT–CT (53%), PET–CT (34%), and Bremsstrahlung SPECT (19%). CT (72%) and MRI (72%) were mostly used for follow-up of tumour status, followed by PET–CT (35%) and PET (2%) (Fig. 4).

**Fig. 9:**
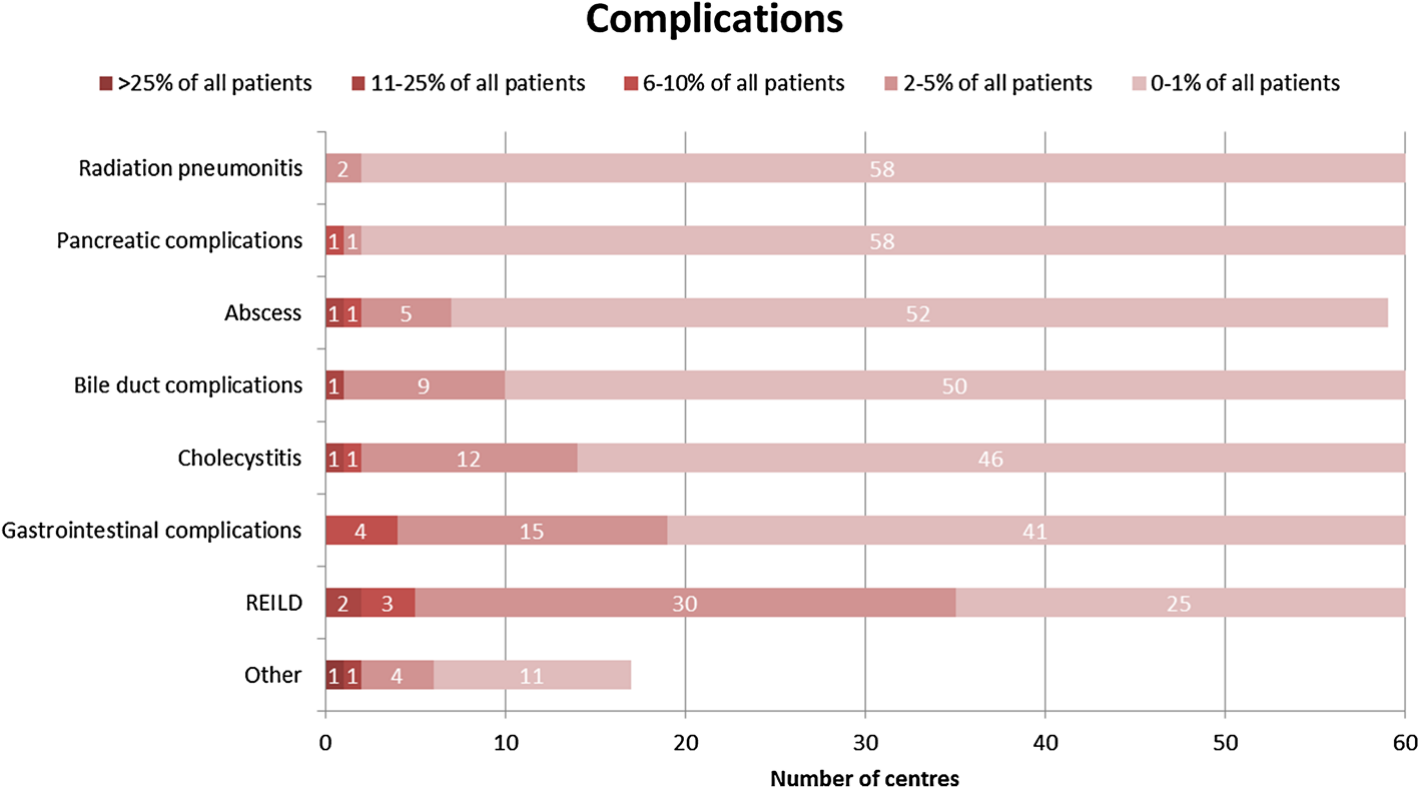
Heat map representing number of centres that encounter complications per patient category (Q23)

The main complications reported after radioembolisation are: radiation pneumonitis, gastrointestinal complications, pancreatic complications, bile duct complications, cholecystitis and abscess, with the centre peak incidences at 0–1% of patients. Radioembolisation induced liver disease (REILD) was more often reported with a peak incidence at 2–5% of patients (Fig. 9).

### Future Developments

**Fig. 10:**
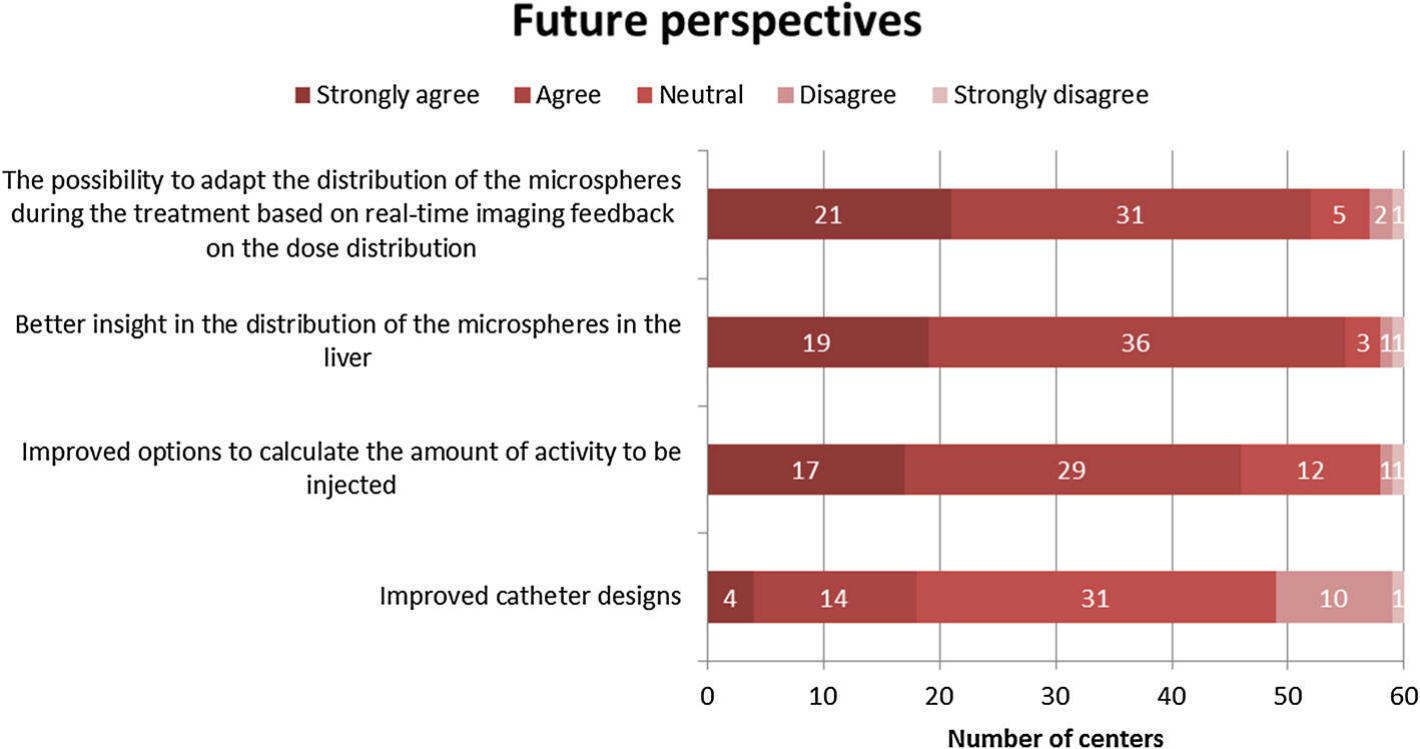
Heat map on future perspectives (Q24)

Most centres agreed that better insights into the distribution of the microspheres in the liver, the possibility to adapt the distribution of the microspheres during the treatment and improved options to calculate the amount of activity to be injected could improve radioembolisation treatment in their practice. There seems to be little enthusiasm for improved catheter designs (Fig. 10). Other improvements proposed by the respondents included improved microsphere administration devices, effective calculation of flow distribution within the liver, and personalised treatment options.

## Discussion

The data from this survey give a unique insight into the current state of radioembolisation in Europe. Overall, a tendency towards an increasing number of radioembolisation procedures is seen which may reflect the way radioembolisation is increasingly recognised as a valuable treatment option in European cancer guidelines. There are still considerable differences between European centres with regard to how radioembolisation is performed.

This is the second time a European survey on radioembolisation practices has been performed [4]. The number of responding centres has risen from 28 in the survey from 2011 to 71 in this survey. This rise is probably the result of the fact that all CIRSE members received an invitation to join the survey, aided by the ease of filling out and returning a digital survey as compared to a survey on paper that had to be returned by regular mail. Also, differences in results may be caused by the fact that the current survey was spread to a different group of centres (all CIRSE members) than the previous survey (list of hospitals found on the microsphere-vendors’ websites).

The rate of response to this survey is unclear since the invitation for this survey was sent to all CIRSE members, approximately 7000 people globally. However, this does not give any insight on the amount of centres that these members are currently working at. The number of centres performing radioembolisation in Europe is probably much higher than 63, but there are no exact figures available. Sixty-three is believed to be a fair number given the fact that responders had to invest quite some time to complete the survey without getting a reward in return.

SIR-Spheres remain the type of microspheres used by most centres, although this share seems to have declined. The fraction of centres using TheraSpheres only has risen, as well as the fraction of centres using both products (Figure S2). What looks like an increase and/or decrease in the use of a certain type of microspheres should be interpreted with caution, as the participating centres are different between surveys. The exact number of treatments per centre and the total amount of treatments performed with SIR-Spheres and TheraSpheres are not clearly determined from this survey.

It is generally accepted that cone-beam CT improves the safety and quality of the radioembolisation procedure in several aspects [5]. Therefore, it would be interesting to know whether the centres that do not use cone-beam CT, do not have a cone-beam CT system available or whether they choose not to use it.

Another interesting finding is that although PET–CT is the most accurate modality for performing ^90^Y-dosimetry, Bremsstrahlung SPECT–CT is still the mainstay for post-treatment ^90^Y-imaging. This may be due to the higher costs and lower availability of PET–CT systems (Figs. 4 and S3).

There is still some debate amongst centres on whether arterio-portal shunting, ascites drainage and extrahepatic diseases should be considered a contraindication or not. Compared to the survey in 2011 not much change is seen in contraindications, except for a shift towards not seeing complete portal vein thrombosis as a contraindication, which was still undecided in 2011.

In accordance with the trend of more selective injection positions, there seems to be a trend towards less coil-embolisation of non-target vessels. A strong decrease is seen in centres that coil-embolise the RGA (50 vs. 59% in 2011), GDA (34 vs. 71% in 2011) and CA (8 vs. 41% in 2011) (Figure S5). Injections from the proper hepatic artery are rare and selective injection in the right- and left liver arteries with a time gap in between is preferred by most centres. Still, it is surprising that 8% of centres regularly coil the cystic artery since there is no evidence that this is beneficial and may even cause ischaemic cholecystitis [6, 7].

When looking at complications, centres are unambiguous, most of the complications are rare and are reported in only 0–1% of the patients. REILD is encountered more frequently, which explains why several groups have studied the occurrence of this complication and ways to optimise activity calculation to prevent REILD [8, 9].

Although the BSA and empirical model to calculate activity are proven suboptimal, there is still a large number of centres that base their decisions on these models with regard to resin microspheres. The more elaborate partition model is still rarely used as this requires more time and physicians may not be comfortable with the high amounts of activity that can result from adhering to this model. Most centres use the MIRD model for activity calculation with glass microspheres.

This survey shows that a significant fraction of patients is excluded or receives a reduced amount of activity based on lung shunt assessment as assessed with ^99m^Tc-MAA. Withholding treatment or reducing the dose may not be necessary in all of these patients since there is evidence that lung shunting is largely overestimated by ^99m^Tc-MAA [10–12]. In particular since there are hardly any cases of radiation pneumonitis reported.

Selection bias could be a limitation of the survey, as centres that perform radioembolisation more often might be more inclined to respond to a survey concerning radioembolisation compared to centres that do not provide radioembolisation as a treatment option in their hospital or only perform a limited number of radioembolisation procedures. Furthermore, it remains unclear whether the presented sample is actually representative for the radioembolisation ‘population’ in Europe. It is also possible that the results would look quite different when taking the USA into account.

In general, the results of this survey show a discrepancy between the techniques that are recommended in the literature and the techniques used in daily practice. Examples are the relatively infrequent use of the partition model, scarce use of Y^90^-PET–CT and cone-beam CT and the high frequency of coil-embolisation. We believe that research initiatives should be aimed at reducing these discrepancies and improving techniques in a way that they can be used by any centre and not only by experts. When asked about future developments, responders indicate that they would benefit from better dosimetry tools to be able to thoroughly evaluate their patients and present them with a more personalised treatment.

## Conclusion

In conclusion, this survey provides insight into the current state of radioembolisation practices across Europe. There is still a large variation between centres in the way radioembolisation is performed, and several trends can be recognised when comparing the results to a previous survey.

## Electronic supplementary material

Below is the link to the electronic supplementary material.

Supplementary material 1 (DOCX 24284 kb)

## Abbreviations

AR: Anti-reflux catheter
Brems.: Bremsstrahlung
(m)BSA: (modified) Body surface area
CIRSE: Cardiovascular Interventional Radiological Society in Europe
CA: Cystic artery
CT: Computed tomography
CTA: Computed tomography angiography
Extra: Extrahepatic deposition assessment
GDA: Gastroduodenal artery
Gy: Gray
HCC: Hepatocellular carcinoma
Intra: Intrahepatic deposition assessment
Lung: Lung shunt assessment
mCRC: Colorectal carcinoma metastases
MIRD: Medical Internal Radiation Dose
MRA: Magnetic resonance angiography
MRI: Magnetic resonance imaging
NA: Not applicable/no answer
PET: Positron emission tomography
RE: Radioembolisation
REILD: Radioembolisation induced liver disease
RGA: Right gastric artery
SPECT: Single-photon emission computed tomography
Std.: Standard catheter
^99m^Tc-MAA: Technetium-99m macroaggregated albumin
UMCU: University Medical Centre Utrecht
URL: Uniform resource locator
USA: United States of America
Y^90^: Yttrium-90
