# Supplementary material for: Radioembolisation in Europe: A Survey Amongst CIRSE Members
Source: Cardiovasc Intervent Radiol. 2018 May 8;41(10):1579–89. doi: 10.1007/s00270-018-1982-4 (PMC6132849; doi:10.1007/s00270-018-1982-4)
Supplement: Supplementary file 1 — Supplementary material 1 (DOCX 24284 kb) [file 270_2018_1982_MOESM1_ESM.docx]

| Country |  | Type | Reason  99m-Tc-MAA | | Lung shunt | Model resin | | Model glass | | Catheter | Injection position | | Regimen  (weeks) | | |
| --- | --- | --- | --- | --- | --- | --- | --- | --- | --- | --- | --- | --- | --- | --- | --- |
| Question |  | 5 | 9 | | 10 | 13a | | 13b | | 16 | 17 | | 18 | | |
| Austria (n=2) | 1 | Resin | Lung + Intra + Extra | | 20% | Partition | | NA | | Std. + AR | Mimic position | | Sequential (4-6) | | |
|  | 2 | Both | Lung + Extra | | 20% | BSA | | MIRD | | Std. + AR | No check | | L/R single | | |
| Belgium (n=3) | 3 | Resin | Lung + Intra + Extra | | 20% | BSA + mBSA | | NA | | Std. + AR | Mimic position | | L/R single | | |
|  | 4 | Resin | Lung | | 20% | BSA | | NA | | Std. | Mimic position | | L/R single | | |
|  | 5 | Resin | Lung + Intra | | 7 Gy | BSA + Partition | | NA | | Std. | Mimic position | | L/R single | | |
| France  (n=5) | 6 | Both | Lung + Intra | | NA | BSA | | MIRD | | Std. | Mimic position | | Sequential (4) | | |
|  | 7 | Both | Lung + Intra + Extra | | 15% | Empirical + Partition | | Empirical | | Std. | Mimic position | | Sequential (4-6) | | |
|  | 8 | Glass | Lung + Intra + Extra | | 15 Gy | NA | | MIRD | | Std. | Mimic position | | Sequential | | |
|  | 9 | Resin | Lung + Intra | | 25 Gy | BSA | | NA | | Std. | Mimic position | | Sequential (4-6) | | |
|  | 10 | Both | Lung + Intra + Extra | | 50 Gy | BSA + Partition | | Other | | Std. | Mimic position | | L/R single | | |
| Germany (n=15) | 11 | Glass | Lung + Intra + Extra | | 30 Gy | NA | | Empirical | | Std. | Mimic position | | Sequential (6) | | |
|  | 12 | Resin | Lung + Intra | | 30 Gy | BSA | | NA | | Std. | Mimic position | | L/R single | | |
|  | 13 | Both | Lung + Intra + Extra | | 20% | Partition | | MIRD | | Std. | Mimic position | | Sequential (4-6) | | |
|  | 14 | Glass | Lung + Intra + Extra | | 30 Gy | NA | | MIRD | | Std. | Mimic position | | Sequential (4) | | |
|  | 15 | Resin | Lung + Intra | | 10% | BSA | | MIRD* | | Std. | Try to recall | | Sequential | | |
|  | 16 | Glass | Lung + Extra | | 15% | NA | | MIRD | | Std. | Mimic position | | Sequential (6) | | |
|  | 17 | Glass | Lung + Intra + Extra | | 20% | NA | | MIRD | | Std. | Try to recall | | Sequential | | |
|  | 18 | Resin | Lung + Intra | | 20% | BSA | | NA | | Std. | Mimic position | | Sequential (4) | | |
|  | 19 | Both | Lung + Intra + Extra | | 10% | BSA | | MIRD | | Std. | Mimic position | | L/R Single | | |
|  | 20 | Both | Lung + Intra + Extra | | 15% | BSA + Partition | | Other | | Std. | Mimic position | | L/R Single | | |
|  | 21 | Resin | Lung + Intra + Extra | | 30 Gy | BSA + mBSA | | NA | | Std. | Mimic position | | Sequential (4-6) | | |
|  | 22 | Resin | Lung + Extra | | 20% | BSA | | NA | | Std. | Mimic position | | Whole liver | | |
|  | 23 | Glass | Lung + Intra | | 20% | NA | | Empirical | | Std. | Mimic position | | L/R Single | | |
|  | 24 | Resin | Intra | | 25% | BSA | | NA | | Std. | Mimic position | | L/R Single | | |
|  | 25 | Resin | Lung + Intra + Extra | | 20% | BSA | | NA | | Std. | Mimic position | | Sequential (4-5) | | |
| Greece (n=1) | 26 | Resin | Lung + Intra + Extra | | 20% | BSA + Partition | | NA | | Std. | Mimic position | | Sequential (4-5) | | |
| Italy  (n=9) | 27 | Both | Lung + Intra + Extra | | 20% | Empirical | | MIRD | | Std. + AR | Mimic position | | Sequential (5) | | |
|  | 28 | Both | Lung + Intra + Extra | | 20% | Partition | | MIRD | | Std. | Mimic position | | Sequential (6-8) | | |
|  | 29 | Both | Lung + Intra + Extra | | 20% | Partition | | Empirical | | Std. | Mimic position | | Sequential | | |
|  | 30 | Both | Lung + Intra | | 25% | BSA + Partition | | MIRD | | Std. + AR | Mimic position | | Sequential (4-6) | | |
|  | 31 | Glass | Lung + Intra + Extra | | 30% | NA | | Empirical + MIRD | | Std. + AR | Mimic position | | Sequential | | |
|  | 32 | Glass | Lung + Intra + Extra | | NA | NA | | Other | | Std. | Mimic position | | Sequential (9) | | |
|  | 33 | Resin | Lung + Intra + Extra | | 10-20 Gy | BSA | | NA | | Std. | Mimic position | | Sequential (4) | | |
|  | 34 | Resin | Lung + Intra + Extra | | 20% | BSA | | NA | | Std. + AR | No check | | Sequential (4) | | |
|  | 35 | Glass | Lung + Extra | | 5% | Empirical + BSA * | | Empirical | | Std. + AR | Mimic position | | Sequential (4) | | |
| Luxem-burg (n=1) | 36 | Resin | Lung + Intra | | 5% | Empirical + BSA + Partition | | NA | | Std. | Mimic position | | Depending on liver function | | |
| Nether- lands  (n=4) | 37 | Glass | Lung + Intra | 15% | | | NA | NA | Std. | | | Mimic position | | L/R Single |  |
|  | 38 | Both | Lung + Extra | 30 Gy | | | Partition | Other | Std. | | | Mimic position | | L/R Single |  |
|  | 39 | Glass | Lung + Intra + Extra | 30 Gy | | | NA | MIRD | Std. | | | Mimic position | | Sequential (4) |  |
|  | 40 | Glass | Lung + Intra + Extra | 20% | | | NA | MIRD | Std. + AR | | | Mimic position | | L/R Single |  |
| Portugal (n=1) | 41 | Resin | Lung + Intra + Extra | 20% | | | BSA | NA | Std. | | | Mimic position | | L/R Single |  |
| Slovenia (n=1) | 42 | Resin | Lung | 10% | | | Empirical | NA | Std. | | | Mimic position | | Sequential (4-6) |  |
| Spain  (n=2) | 43 | Glass | Lung + Intra + Extra | 20% | | | NA | MIRD | Std. | | | Mimic position | | L/R Single |  |
|  | 44 | Glass | Lung + Intra + Extra | NA | | | Partition * | MIRD | Std. | | | Mimic position | | Sequential |  |
| Sweden  (n=1) | 45 | Resin | Lung | 5% | | | BSA + Partition | NA | Std. | | | Mimic position | | L/R Single |  |
| Switzer-land (n=4) | 46 | Resin | Lung | 50% | | | Empirical | Empirical * | Std. | | | No check | | L/R Single |  |
|  | 47 | Resin | Lung + Intra + Extra | 20% | | | BSA | NA | Std. | | | Mimic position | | L/R Single |  |
|  | 48 | Resin | Lung + Intra | NA | | | BSA | NA | Std. | | | Mimic position | | Sequential (4-6) |  |
|  | 49 | Resin | Lung | 25% | | | BSA | NA | Std. | | | Mimic position | | Whole liver |  |
| Turkey  (n=6) | 50 | Both | Lung + Intra + Extra | 20% | | | BSA + Partition + mBSA | MIRD | Std. | | | Mimic position | | Sequential (4) |  |
|  | 51 | Both | Lung + Intra + Extra | 20% | | | Partition | MIRD | Std. | | | Fluoro-scopically | | Sequential (4) |  |
|  | 52 | Both | Lung + Intra | 20% | | | BSA | Empirical | Std. | | | Mimic position | | Sequential (3) |  |
|  | 53 | Glass | Lung + Intra + Extra | 20% | | | NA | MIRD | Std. | | | Mimic position | | Sequential (4) |  |
|  | 54 | Resin | Lung + Intra + Extra | 30% | | | NA | NA | Std. | | | Mimic position | | Sequential (4) |  |
|  | 55 | Glass | Lung + Intra + Extra | 20% | | | NA | Other | Std. | | | Mimic position | | L/R Single |  |
| United Kingdom (n=5) | 56 | Both | Lung + Intra | 30% | | | BSA | MIRD | Std. | | | Mimic position | | L/R Single |  |
|  | 57 | Both | Lung + Intra | 30 Gy | | | BSA + Partition | Empirical | Std. | | | Mimic position | | Other |  |
|  | 58 | Both | Lung + Intra + Extra | 20% | | | BSA | MIRD | Std. | | | Mimic position | | Whole liver |  |
|  | 59 | Both | Lung + Intra | 20% | | | BSA | Empirical | Std. | | | Mimic postion | | L/R Single |  |
|  | 60 | Both | Lung + Intra + Extra | 20% | | | BSA | Empirical | Std. | | | Mimic position | | L/R Single |  |
| Total |  | Resin 40% / Glass 27% / Both 33% | Lung 98% / Intra 83% / Extra 65% | Avg. 17.5% | | | Empirical 9% / BSA 58% / Partition 28% / mBSA 5% | Empirical 28% / MIRD 58% / Other 14% | Std 100% / AR 15% | | | Mimic position 92% / Try to recall position 3% / No check 5% | | Sequential 55% / Single session 38% / Whole liver 5% / Other 2% |  |

**Table S1** Answers of centres participating in the survey

Lung = Lung shunt assessment, Intra = Intrahepatic deposition assessment, Extra = extrahepatic deposition assessment, NA = Not applicable/No answer, * = not in accordance with answer to question 5, (m)BSA = (moderated) Body Surface Area Model, MIRD = Medical Internal Radiation Dose Model, Std. = Standard catheter, AR = anti reflux catheter

| Country |  | Pre-treatment Imaging | Art. liver anatomy | Evaluation  99m-Tc-MAA | C-arm  (Cone-beam)  CT | Post-treatment imaging | FU Imaging |
| --- | --- | --- | --- | --- | --- | --- | --- |
| Question |  | 6a | 6b | 8 | 19 | 20 | 22 |
| Austria (n=2) | 1 | CT + MRI | None | SPECT | Extra + Tumour | 90Y Brems SPECT-CT | CT + MRI |
|  | 2 | MRI | CTA | SPECT | Not used | 90Y Brems SPECT | CT |
| Belgium (n=3) | 3 | MRI | None | SPECT-CT | Extra + Tumour | 90Y Brems SPECT-CT | MRI + PET-CT |
|  | 4 | CT | None | SPECT-CT | Tumour + Volume | 90Y Brems SPECT-CT | CT + MRI + PET-CT |
|  | 5 | CT + MRI + PET-CT | CTA | SPECT-CT | Extra + Tumour | 90Y PET-CT | CT + MRI + PET-CT |
| France (n=5) | 6 | CT + MRI + PET-CT | CTA | SPECT-CT | Extra + Tumour | 90Y Brems SPECT-CT | MRI |
|  | 7 | CT + MRI + PET-CT | CTA | SPECT + SPECT-CT | Extra + Tumour + Volume | 90Y PET-CT | MRI |
|  | 8 | CT + MRI | CTA | SPECT-CT | Extra + Tumour | 90Y Brems SPECT-CT | MRI |
|  | 9 | CT + MRI + PET-CT | CTA | Planar + SPECT-CT | Extra + Tumour + Volume | 90Y PET-CT +  90Y Brems SPECT-CT | CT + MRI + PET-CT |
|  | 10 | CT + MRI | CTA | SPECT-CT | Extra + Tumour + Volume | 90Y PET-CT | CT + MRI |
| Germany (n=15) | 11 | MRI | MRA | SPECT-CT | Not used | 90Y PET-CT | MRI |
|  | 12 | CT + MRI | CTA | SPECT | Extra + Tumour + Volume | 90Y Brems SPECT-CT | MRI |
|  | 13 | CT + MRI + PET-CT | MRA | SPECT | Extra + Tumour | 90Y Brems SPECT | CT + MRI |
|  | 14 | CT | CTA | SPECT-CT | Extra + Tumour + Volume | 90Y Brems SPECT-CT | CT |
|  | 15 | MRI + PET-CT | MRA | SPECT-CT | Tumour + Volume | 90Y PET-CT | MRI |
|  | 16 | CT + MRI | CTA | SPECT-CT | Tumour | 90Y Brems SPECT-CT | CT |
|  | 17 | CT + MRI | CTA | SPECT | Not used | 90Y Brems SPECT | MRI |
|  | 18 | MRI + PET-CT | None | SPECT | Extra | 90Y PET-CT +  90Y Brems SPECT | PET-CT |
|  | 19 | CT + MRI | CTA | SPECT | NA | 90Y PET-CT | CT + MRI |
|  | 20 | MRI + PET-CT | MRA | SPECT-CT | Extra + Tumour | 90Y PET-CT | MRI + PET-CT |
|  | 21 | CT + MRI | CTA | SPECT-CT | Extra + Tumour + Volume | NA | CT + MRI |
|  | 22 | MRI + PET-CT | CTA | SPECT-CT | Extra | None | MRI |
|  | 23 | PET-CT | CTA | SPECT-CT | Extra + Tumour | 90Y Brems SPECT-CT | CT + MRI+ PET-CT |
|  | 24 | PET-CT | CTA | SPECT-CT | Not used | 90Y Brems SPECT | CT + MRI |
|  | 25 | CT + MRI | CTA | SPECT | Not used | 90Y Brems SPECT | CT + MRI |
| Greece (n=1) | 26 | MRI | None | SPECT-CT | Tumour | 90Y Brems SPECT +  90Y Brems SPECT-CT | CT + MRI |
| Italy (n=9) | 27 | PET-CT | CTA | SPECT-CT | Extra + Tumour + Volume | 90Y Brems SPECT-CT | MRI |
|  | 28 | CT + PET-CT | CTA | Planar + SPECT-CT | Not used | 90Y Brems SPECT | CT + PET-CT |
|  | 29 | CT + MRI | None | SPECT-CT | NA | 90Y Brems SPECT | CT + MRI |
|  | 30 | CT + MRI + PET-CT | CTA | NA | Extra + Tumour | 90Y Brems SPECT +  90Y Brems SPECT-CT | CT + PET-CT |
|  | 31 | CT + MRI + PET-CT | CTA | SPECT + SPECT-CT | Tumour + Volume | 90Y Brems SPECT-CT | CT + MRI |
|  | 32 | CT + MRI | CTA | SPECT-CT | Tumour | 90Y PET-CT +  90Y Brems SPECT-CT | CT + MRI |
|  | 33 | CT + MRI + PET-CT | CTA | SPECT-CT | Extra + Tumour + Volume | 90Y PET-CT | CT + PET-CT |
|  | 34 | CT +  PET-CT | CTA | SPECT-CT | Tumour | 90Y PET-CT | CT + MRI |
|  | 35 | CT | CTA | SPECT-CT | Not used | 90Y Brems SPECT-CT | CT + MRI |
| Luxem-burg (n=1) | 36 | CT + MRI + PET-CT | CTA | SPECT-CT | NA | 90Y Brems SPECT + 90Y Brems SPECT-CT | CT + MRI + PET + PET-CT |
| Nether-lands (n=4) | 37 | CT | CTA | SPECT-CT | Extra + Tumour | 90Y PET-CT | CT |
|  | 38 | CT | CTA | SPECT-CT | Extra + Tumour + Volume | 90Y Brems SPECT-CT | CT |
|  | 39 | MRI | CTA | SPECT-CT | Extra + Tumour + Volume | 90Y Brems SPECT-CT | MRI |
|  | 40 | CT +  PET-CT | CTA | Planar + SPECT-CT | Extra + Tumour + Volume | 90Y PET-CT | CT + PET-CT |
| Portugal (n=1) | 41 | CT +  PET-CT | None | SPECT-CT | Not used | 90Y Brems SPECT-CT | CT + PET-CT |
| Slovenia (n=1) | 42 | MRI | CTA | SPECT-CT | Tumour | None | MRI |
| Spain (n=2) | 43 | CT + MRI | CTA | Planar + SPECT-CT | Not used | 90Y PET-CT | CT + MRI |
|  | 44 | CT + MRI + PET-CT | CTA | SPECT | Tumour + Volume | None | CT + MRI + PET-CT |
| Sweden (n=1) | 45 | CT + MRI + PET-CT | None | SPECT | Not used | 90Y Brems SPECT | CT + MRI |
| Switzer-land (n=4) | 46 | CT + MRI + PET-CT | CTA | Planar | Tumour | None | CT + MRI + PET-CT |
|  | 47 | CT +  PET-CT | CTA | SPECT-CT | Extra + Tumour | 90Y Brems SPECT-CT | CT + PT-CT |
|  | 48 | CT | None | SPECT-CT | Extra | 90Y Brems SPECT-CT | CT |
|  | 49 | CT + MRI + PET-CT | CTA | SPECT-CT | Extra + Tumour | 90Y Brems SPECT-CT | CT + MRI + PET-CT |
| Turkey (n=6) | 50 | MRI + PET-CT | None | SPECT-CT | Extra + Tumour + Volume | 90Y Brems SPECT-CT | MRI + PET-CT |
|  | 51 | CT + MRI + PET-CT | CTA | SPECT-CT | Extra + Tumour | 90Y Brems SPECT + 90Y Brems SPECT-CT | CT + MRI + PET-CT |
|  | 52 | CT + MRI + PET-CT | None | SPECT | Extra + Tumour | 90Y Brems SPECT | CT + MRI + PET-CT |
|  | 53 | MRI + PET-CT | None | SPECT-CT | Tumour | 90Y Brems SPECT-CT | MRI |
|  | 54 | CT +  PET-CT | CTA | SPECT-CT | Not used + Tumour * | 90Y Brems SPECT-CT | CT + PET-CT |
|  | 55 | CT + MRI | CTA | SPECT | Not used | 90Y Brems SPECT-CT | CT + MRI + PET-CT |
| United Kingdom (n=5) | 56 | CT + MRI | None | SPECT-CT | Not used | 90Y PET-CT | MRI |
|  | 57 | CT | CTA | SPECT-CT | Volume | 90Y Brems SPECT-CT | CT |
|  | 58 | CT + MRI | CTA | SPECT-CT | Extra + Volume | 90Y Brems SPECT-CT | CT |
|  | 59 | CT | CTA | SPECT-CT | Tumour | 90Y Brems SPECT-CT | CT |
|  | 60 | CT + MRI | CTA | SPECT-CT | Extra + Tumour | 90Y Brems SPECT-CT | CT + MRI |
| Total |  | CT 75% / MRI 70% / PET-CT 50% | None 22% / CTA 72% / MRA 7% | Planar 8% / SPECT 23% / SPECT-CT 77% | Not used 22% / Extra 52% / Tumour 67% / Volume 30% / Other 3% | None 7% / 90Y PET-CT 34% / 90Y Brems SPECT 19% / 90Y Brems SPECT-CT 53% | CT 72% / MRI 72% / PET 2% / PET-CT 37% |

**Table S2** Answers of centres participating in the survey regarding imaging

CT = computed tomography, MRI = magnetic resonance imaging, PET-CT = positron emission tomography-computed tomography, CTA = computed tomography angiography, MRA = magnetic resonance angiography, SPECT = single-photon emission computed tomography, SPECT-CT = single-photon emission computed tomography - computed tomography, Extra = Extrahepatic deposition assessment, Tumour = to check tumour coverage, Volume = for volumetric analysis, 90Y = Yttrium 90, Brems = Bremsstrahlung

# Supplemental Figures

# Appendix


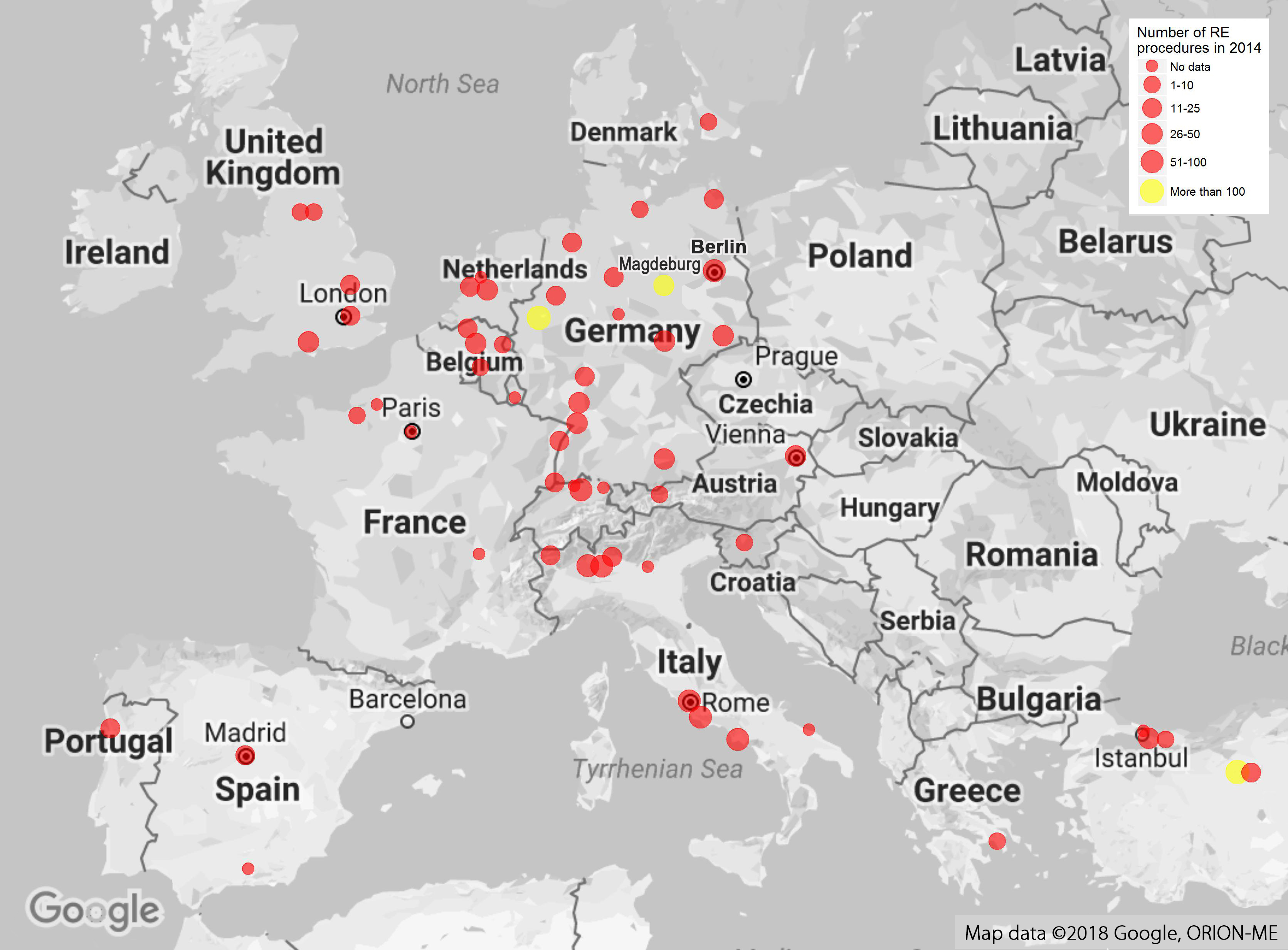

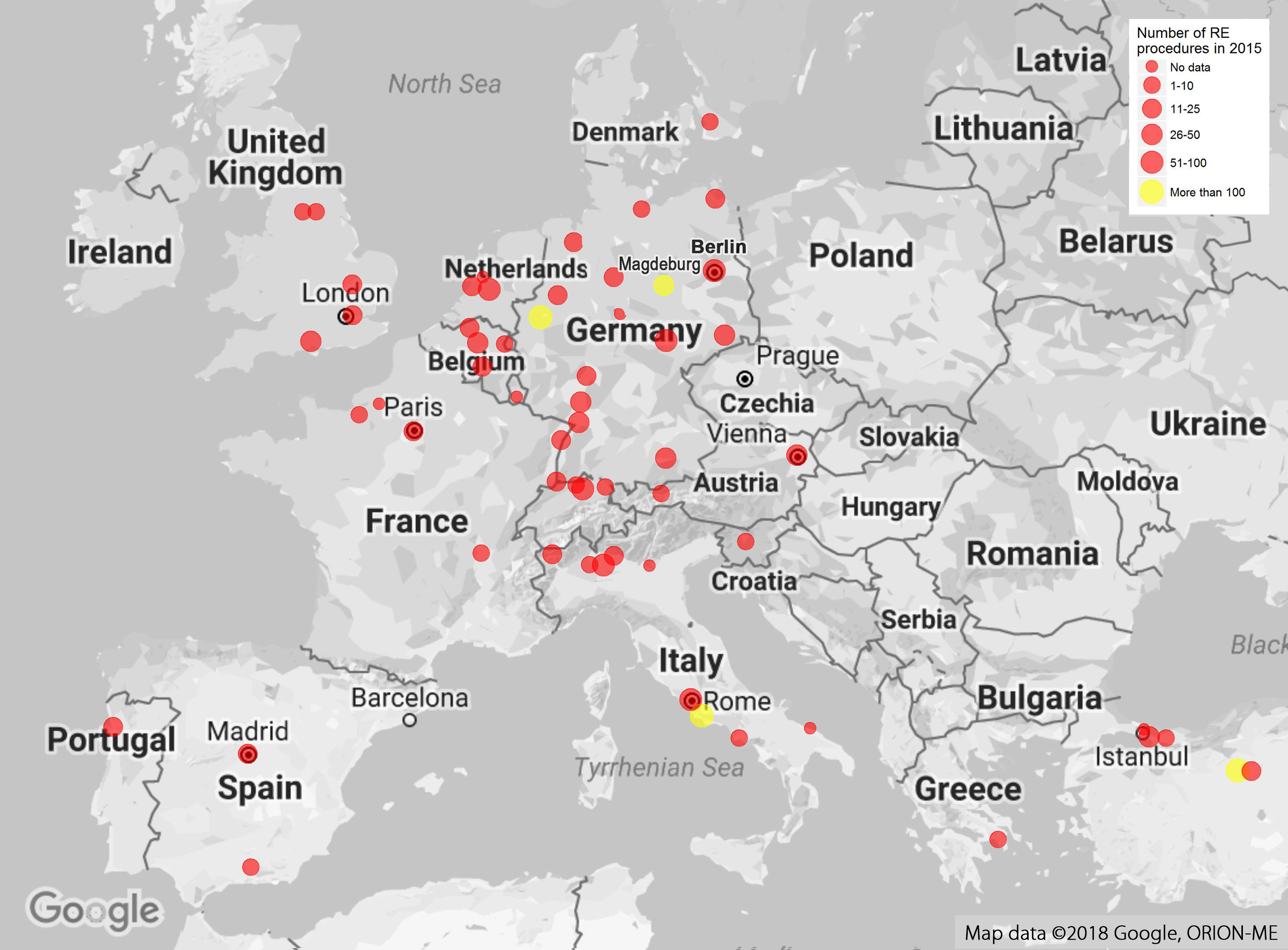


**Supplemental Figure 1a** Geographical representation of number of radioembolisation procedures per centre in 2014 (Q3)

**Supplemental Figure 1b** Geographical representation of number of radioembolisation procedures per centre in 2015 (Q3)


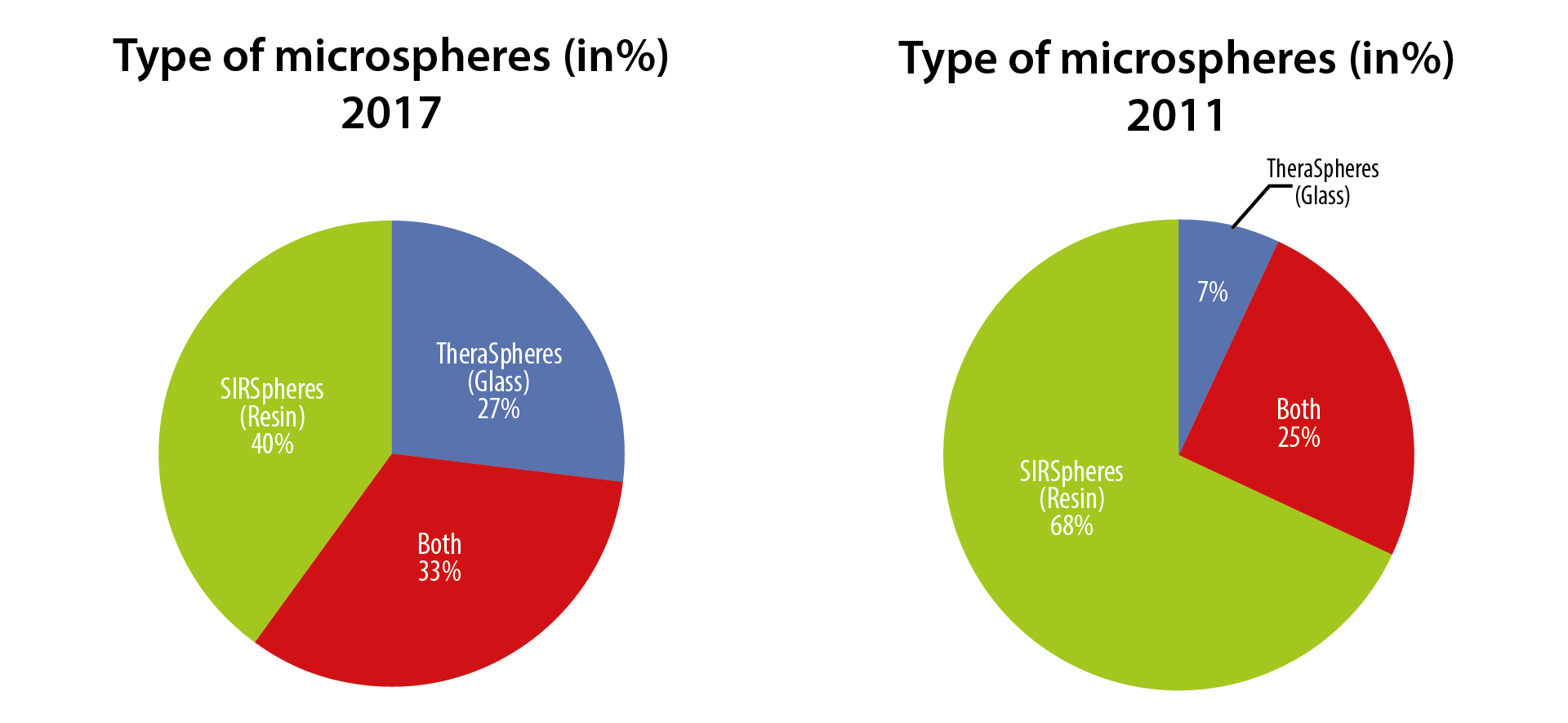


**Supplemental Figure 2a** Percentage of type of microspheres used among participating centres 2017 vs. 2011 (Q5)


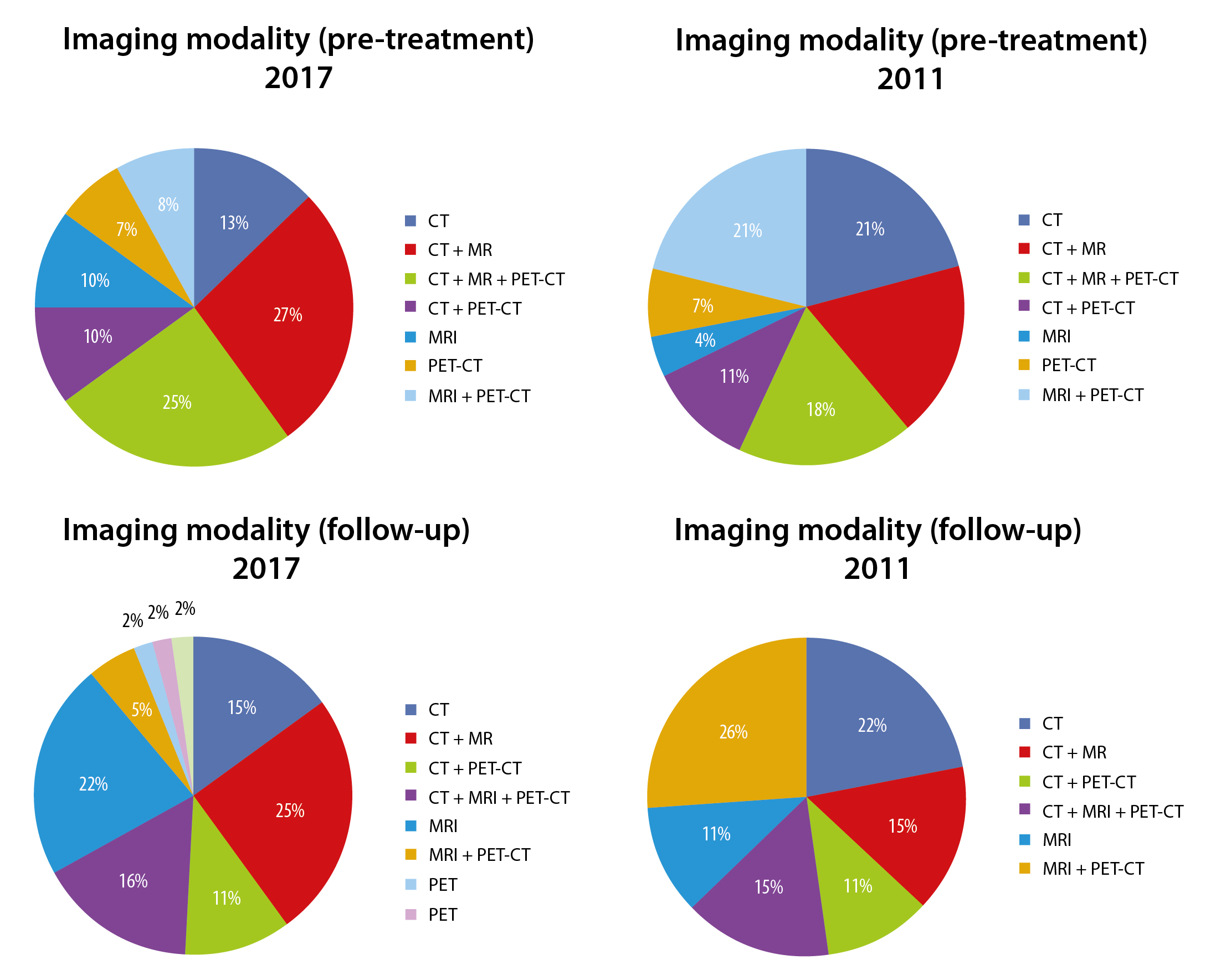
 **Supplemental Figure 3** Imaging modalities 2017 vs. 2011 (Q6a and Q22)


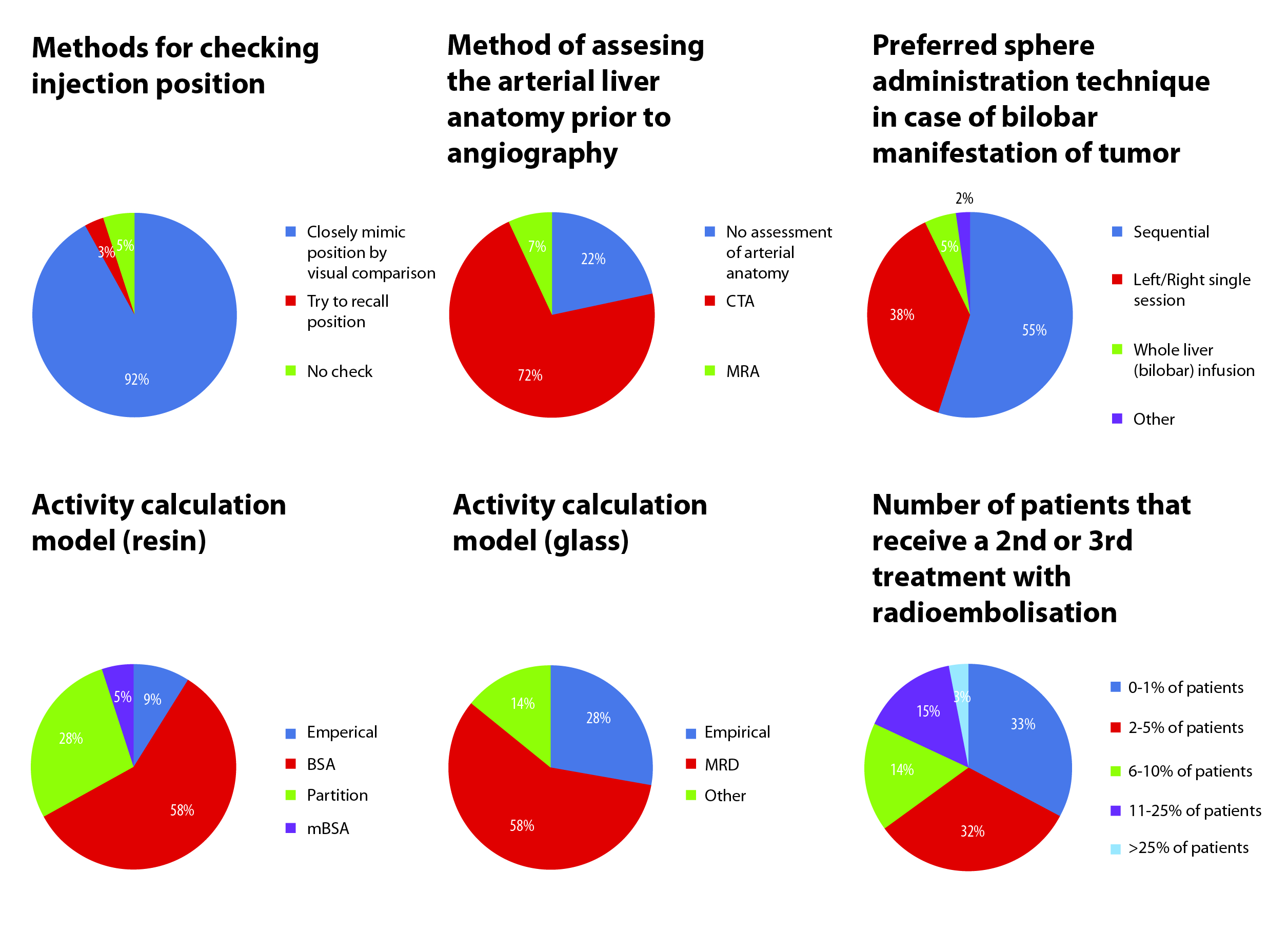


**Supplemental Figure 4** Pie charts Upper panel (left-right) regarding questions (Q6b, Q13a, Q13b, Q17, Q18 and Q21)


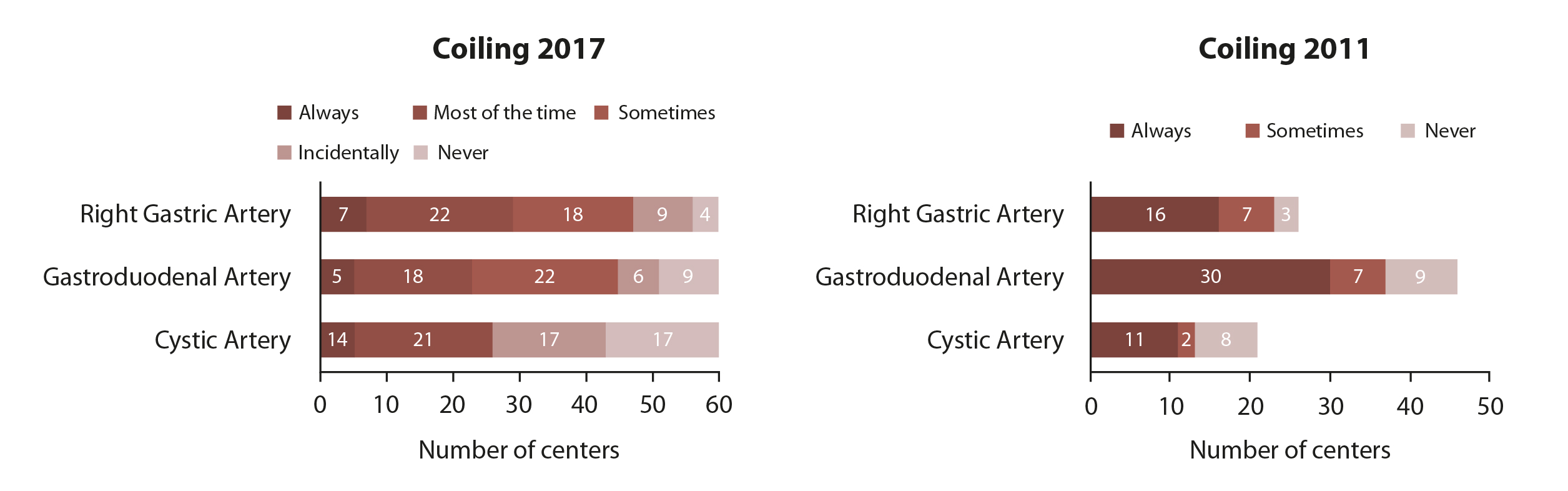


**Supplemental Figure 5** Heat maps regarding coiling 2017 vs. 2011 (Q14)
